# Effects of Holder pasteurization on the protein profile of human milk

**DOI:** 10.1186/s13052-016-0248-5

**Published:** 2016-04-07

**Authors:** Chiara Peila, Alessandra Coscia, Enrico Bertino, Maria Cavaletto, Stefano Spertino, Sara Icardi, Claudia Tortone, Gerard H. A. Visser, Diego Gazzolo

**Affiliations:** Department of Public Health and Pediatrics, Neonatology Unit, University of Turin, Via Ventimiglia, 3, I-10131 Turin, Italy; Department of Science and Technological Innovation, DiSIT, University of East Piedmont “A. Avogadro”, Alessandria, Italy; Department of Obstetrics, University Medical Center, Utrecht, The Netherlands; Department of Maternal, Fetal and Neonatal Health, C. Arrigo Children’s Hospital, Alessandria, Italy

**Keywords:** Human milk, Donor human milk, Pasteurization holder, GeLC-MS, Tenascin

## Abstract

**Background:**

The most widespread method for the treatment of donor milk is the Holder pasteurization (HoP). The available literature data show that HoP may cause degradation of some bioactive components. The aim of this study was to determine the effect of HoP on the protein profile of human milk (HM) using a GeLC-MS method, a proteomic approach and a promising technique able to offer a qualitative HM protein profile.

**Methods:**

HM samples were collected by standardized methods from 20 mothers carrying both preterm and term newborns. A aliquot of each sample was immediately frozen at -80 °C, whilst another one was Holder pasteurized and then frozen. All samples were then analyzed by GeLC-MS. The protein bands of interest were excised from the gel, digested with trypsin and identified by nano-HPLC-MS/MS analysis.

**Results:**

The protein profile before and after HoP showed qualitative differences only in 6 samples out of 20, while in the remaining 14 no detectable differences were found. The differences interested only colostrums and transitional milk samples and regarded the decrease of the electrophoretic bands corresponding to alpha and beta-casein, tenascin, lactoferrin and immunoglobulin.

**Conclusions:**

In the majority of samples, HoP did not cause any modification, thereby preserving the biological activity of HM proteins.

## Background

Human milk (HM) is considered the “gold standard” nutrition for feeding term and pre-term newborns [1]. When mother’s own milk is not available or not sufficient, despite significant lactation support, Donor Human Milk (DM) is an important and the first alternative, especially in high risk newborns admitted to Neonatal Intensive Care Units (NICU) [2, 3].

The main benefit deriving from the use of DM versus formula milks, in preterm infant feeding, is the reduction in the incidence of necrotizing enterocolitis (NEC) as shown by several meta-analysis [4–7]. Moreover, an enhanced feeding tolerance has also been reported [3, 8].

The DM must be collected, processed and stored ensuring its microbiological safety maintaining at the same time the nutritional quality (NQ) [9]. NQ control of HM storage is complex as well as mandatory and pasteurization procedure is the main processing step to inactivate pathogenic microorganisms [2, 10].

DM is typically pasteurized by Holder Pasteurization method (HoP) characterized by heating at 62.5 °C for 30 min. HoP is currently the requested procedure by the majority of Human Milk Banking Associations (HMBA), being a temperature below 62,5 °C considered not safe [2, 10].

The impact of HoP on the biological quality of HM has been investigated and results are still controversial and matter of debate. The main explanations reside in the different procedures regarding samples collection, storage and treatment [11–13]. In this regard, data on the total protein count and specific proteins have been provided by using immune-enzymatic techniques such as ELISA. Recently, GeLC-MS, a proteomic approach involving the separation of proteins in SDS-PAGE by one-dimensional electrophoresis (1DE) followed by identification by mass spectrometry (MS), has been suggested as a promising technique able to offer a qualitative HM protein profile [14]. Data on GeLC-MS pattern in HM previously treated by HoP and later on stored at Human Milk Bank (HMB) are still lacking.

Therefore, the purpose of the present study was to determine the effect of HoP on the protein profile of HM by using GeLC-MS analysis, under reducing and non-reducing conditions, in order to investigate the qualitative HM protein profile and the occurrence of protein aggregates, eventually formed after heat treatment.

## Methods

### Sample collection

We conducted a pretest–test design, where HM samples acted as their own controls. Breast milk was collected, at different stage of maturation (colostrum: *n* = 9; transitional milk: *n* = 5; mature milk: *n* = 6) according to Playford et al. [15], from 20 mothers delivered between 23 and 41 weeks of gestational age (GA). The study was approved by local ethic committee and mother gave informed and signed consent to the study.

Exclusion criteria were: maternal infections, tobacco smokers, drugs addiction and alcoholic; use of drugs or pharmacologically active substances; mothers who received blood transfusions or blood products, or organ transplants; fetal malformations, chromosomal abnormalities, perinatal asphyxia and dystocia.

HM samples were collected at two consecutive mornings, between 8 and 9 a.m., into disposable high density polyethylene sealed bottles (Flormed, Napoli, Italy) sterilized by using ethylene oxide. Milk expression was obtained by emptying one or two breasts with an electric breast pump (Medela Symphony). From each container, 10 mL of HM were taken, divided into two fractions: the first was immediately frozen at − 80 °C; the second was pasteurized in HMB and frozen at − 80 °C. HoP was performed with a Sterifeed Pasteuriser by Medicare Colgate Ltd (Cullompton, England), heating milk at 62.5 °C for 30 min. The last HoP phase requires a rapid and precise cooling of milk samples to 10 °C in approximately 20 min, by immersion into cold water.

### Sample preparation and protein quantification

Skimmed HM samples were obtained by centrifugation at 2000 × g for 30 min at 10 °C, the pellet and the floating layer were discarded. Protein content was estimated according to Bradford [16].

### GeLC-MS analysis and protein identification

Skimmed milk samples were mixed with Laemmli buffer (2 % w/v SDS, 10 % Glycerol, 5 % β-mercaptoethanol, 62 mM Tris-HCl pH 6.8), boiled for 5 min, and loaded on 10 × 8 cm vertical 12 % polyacrylamide gels. For non-reducing conditions the Laemmli buffer did not contain β-mercaptoethanol and samples were not boiled.

SDS-PAGE was performed at 10 mA per gel for 30 min and 30 mA per gel until the tracking dye front reached the bottom of the gel, at 10 °C with a Mini Protean II Xi System (Bio-Rad). The running buffer was 25 mM Tris-HCl, 200 mM Glycine, 0.1 % w/v SDS. The gels were stained overnight with Colloidal Coomassie brilliant blue G250 (Bio-Rad Laboratories) in accordance with Neuhoff et al. [17]. The Coomassie-stained gels were scanned using an Image Scanner III (GE Healthcare) at 300 dpi. The protein bands of interest were manually excised from 1DE gels and in-gel digested with trypsin as described by Spertino et al. [18]. The peptide mixtures were pooled and lyophilized in a SpeedVac for mass spectrometry analysis.

MS/MS analysis was performed using a QSTAR XL hybrid quadrupole-TOF instrument (Applied Biosystems, Foster City, CA, USA) coupled with a LC Packings Ultimate 3000 nano-flow LC system (Dionex, Amsterdam, The Netherlands), as described by Bona et al. [19]. Briefly, the QSTAR XL operated in positive mode and in information-dependent acquisition (IDA) mode, the dynamic exclusion feature of the Analyst QS 1.1 software (Applied Biosystems, Foster City, CA, USA) was enabled, with an exclusion mass width of +/−3 m/z for 60 s. LC/MS-MS files obtained from each protein sample were merged into a single MASCOT generic format (mgf) file and searched against the NCBI non-redundant database; tolerance for precursor and fragment masses was 0.25 Da. The proteins were identified in homology with significant ion scores (*p* < 0.05).

### Statistical analysis

Clinical data are reported as the mean and SD. Protein content (mg/mL) are reported as median and interquartile ranges. Statistical analysis was performed using XLStat-Pro v.7.2.5 (Addinsoft, New York, USA). Results were compared between groups by Mann-Whitney U-two sided test when the data did not follow a Gaussian distribution. A value of *P* < 0.05 was considered significant.

## Results

### Demographic characteristics of milk donors

The demographic characteristics of the milk donors are shown in Table 1. As expected, the incidence of delivery mode and the need of caesarean section were within the reference for our country. Gestational age and maternal age at birth were within reference curve for our national standards. All mothers showed normal clinical conditions. No overt neurological injury and/or infections were observed at the sampling time-points or at discharge from the hospital.

**Table 1:** Demographic characteristics of milk donors

Donor	Mother’s ethnic group	Age (yrs)	Previous breastfeeding	GA (wks)	Delivery mode	Milk maturity degree	Spontaneous pregnancy
1	Caucasian	38	Yes	28	CS	Mature	Yes
2	Caucasian	31	Yes	25	V	Transitional	Yes
3	Caucasian	38	Yes	35	C	Transitional	Yes
4	Caucasian	35	Yes	31	CS	Mature	Yes
5	Caucasian	35	No	23	CS	Mature	No
6	Caucasian	39	No	35	V	Transitional	Yes
7	Caucasian	37	No	27	V	Transitional	Yes
8	Caucasian	25	No	29	CS	Mature	Yes
9	Caucasian	31	Yes	32	CS	Mature	Yes
10	Caucasian	33	No	33	CS	Transitional	Yes
11	Caucasian	30	No	31	CS	Mature	No
12	Caucasian	22	No	38	V	Colostrum	Yes
13	Other	33	Yes	40	V	Colostrum	Yes
14	Caucasian	28	No	38	V	Colostrum	Yes
15	Caucasian	34	No	39	CS	Colostrum	Yes
16	Caucasian	40	Yes	38	V	Colostrum	Yes
17	Caucasian	39	Yes	37	V	Colostrum	Yes
18	Caucasian	36	No	37	V	Colostrum	Yes
19	Caucasian	37	Yes	37	V	Colostrum	Yes
20	Caucasian	33	No	41	V	Colostrum	Yes

*Abbreviations*: yrs Years, *wks* weeks, *CS* caesarean section, *V* vaginal

### Total protein content determination

The data of protein content are reported in No-Holder pasteurized (NO-HoP) and Holder pasteurized (HoP) groups in Tables 2 and 3. Both in NO-HoP than in HoP groups no significant differences (*P* > 0.05, for all) have been found in total protein content, also after correction for milk maturity degree (Table 2).

**Table 2:** The median of protein content in human milk samples before (No-HoP) and after Holder pasteurization (HoP)

Parameters	No-HoP			HoP		
	Median	25 %	75 %	Median	25 %	75 %
All milk samples (*n* = 20)	14.49	10.28	17.22	11.96	10.21	15.65
Colostrum (*n* = 9)	14.58	13.69	20.32	12.22	11.32	17.72
Transitional (*n* = 5)	14.89	10.42	16.76	14.50	10.63	15.52
Mature (*n* = 6)	11.30	8.91	16.71	9.96	8.35	12.00

Data are given in median and interquartile ranges

**Table 3:** Protein content (mg/ml) in human milk samples before (No-HoP) and after Holder pasteurization (HoP)

Sample	No- HoP	HoP
1	12,99	10,16
2	9,99	10,76
3	17,85	14,50
4	8,92	12,00
5	8,02	7,62
6	14,89	15,39
7	10,57	10,27
8	16,71	8,36
9	29,10	30,13
10	16,40	15,92
11	9,63	9,77
12	29,42	21,87
13	13,09	7,82
14	14,59	11,11
15	13,89	12,23
16	14,41	11,92
17	16,29	15,14
18	28,05	31,86
19	9,83	11,40
20	17,74	16,34

### Proteomic analysis and protein identification

**Fig. 1:**
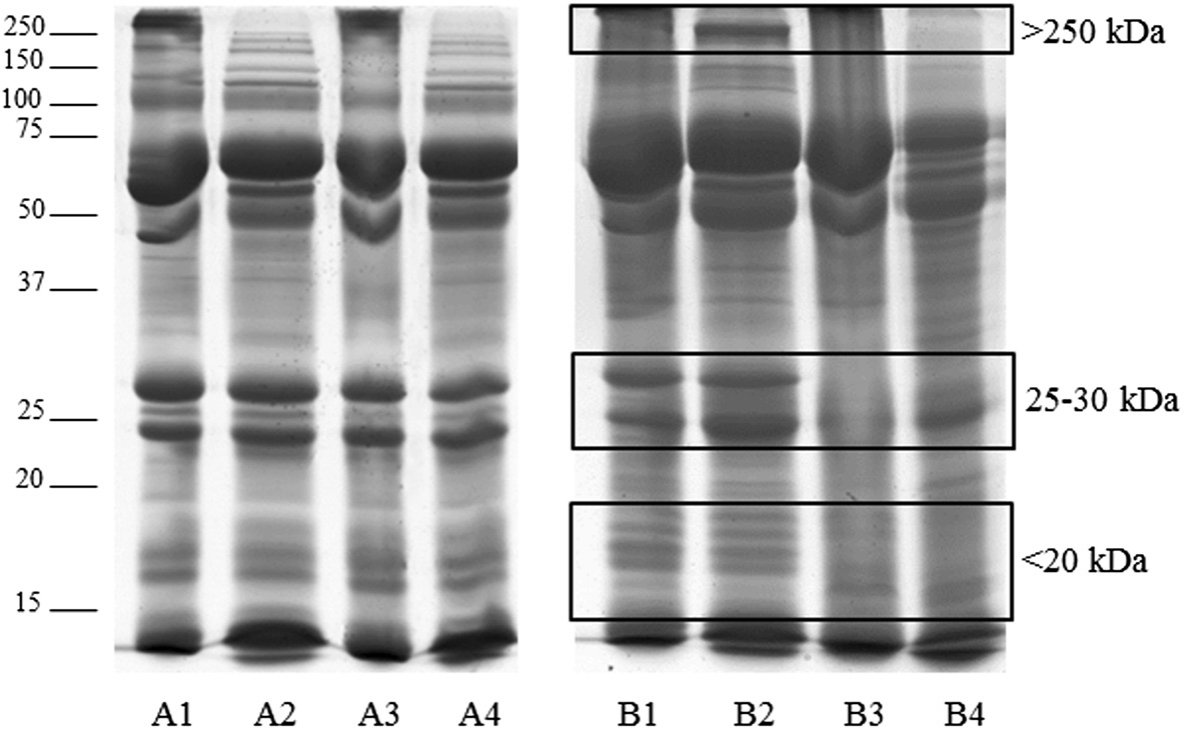
Representative 1DE profiles of pasteurized milks. Legend. Sample A: milk unchanged after Holder pasteurization; sample B: milk changed after Holder pasteurization. For each sample, lane 1: raw milk under non-reducing conditions; lane 2: raw milk under reducing conditions; lane 3: pasteurized milk under non-reducing conditions; lane 4: pasteurized milk under reducing conditions. Differences in band profiles were observed in three molecular weight regions, indicated by the squares

The separation of proteins using 1DE permits to visualize the various protein species in a biological sample; Fig. 1 shows electrophoretic separation of milk’s proteins, under reducing and non-reducing conditions.

**Fig. 2:**
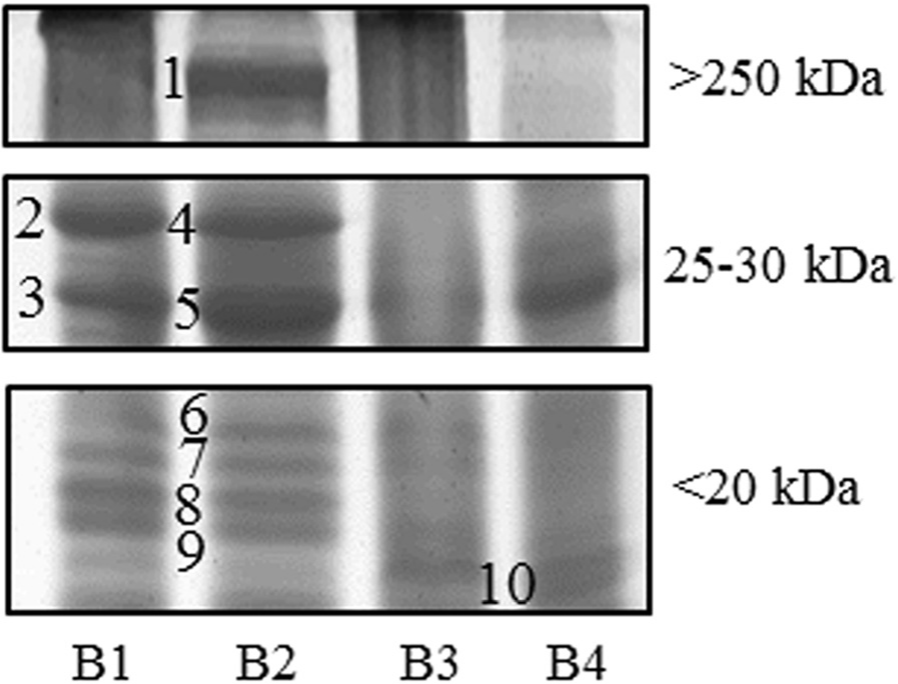
1DE detail of changed protein bands in the different molecular weight regions. Legend. Numbers indicate the protein bands identified by MS/MS analysis

Six out of 20 samples, corresponding to colostrum (*n* = 5) and transitional milk (*n* = 1), showed a reduction of band intensity whilst no changes were observed in the mature milk samples. There were no differences in the six samples after correction for delivery mode and/or gestational age at birth. Reduction of intensity was observed in three regions: <20 kDA; 25-30 kDa; >250 kDa. Concerning the bands in the region between 25 and 30 kDa, the distribution of aggregates between protein fragments was altered by pasteurization in five milk samples, individually or in association with other modifications. The proteins identified by MS in the different regions (Fig. 2), are shown in Table 4.

**Table 4:** List of proteins identified from bands showing changes after Holder pasteurization

Band no.	Protein ID	AC number (gi NCBI)	Theoretical Mr (kDa)	MASCOT score	Sequence coverage (%)	MS/MS sequencing
1	TNC variant protein	68533131	244.2	1177	15 %	R.LEELENLVSSLR.E
						K.FTTDLDSPR.D
						R.ELEPGVEYFIR.V
						R.VFAILENKK.S
						R.VATYLPAPEGLK.F
						R.QTGLAPGQEYEISLHIVK.N
						R.LDAPSQIEVK.D
						K.ETFTTGLDAPR.N
						R.VSQTDNSITLEWR.N
						K.TTLTGLRPGTEYGIGVSAVK.E
						K.EDKESNPATINAATELDTPK.D
						K.ESNPATINAATELDTPK.D
						R.GLEPGQEYNVLLTAEK.G
						K.AATPYTVSIYGVIQGYR.T
						R.AVDIPGLEAATPYR.V
						R.TPVLSAEASTAK.E
						K.QSEPLEITLLAPER.T
						R.EATEYEIELYGISK.G
						R.APTAQVESFR.I
						K.FTTDLDSPR.D
						R.DLTATEVQSETALLTWRPPR.A
						K.EVIVGPDTTSYSLADLSPSTHYTAK.I
						K.IQALNGPLR.S
						R.EEFWLGLDNLNK.I
						K.ITAQGQYELR.V
						R.DHGETAFAVYDKFSVGDAK.T
2	Beta-casein	288098	25.2	63	12 %	K.SPTIPFFDPQIPK.L
						K.VLPIPQQVVPYPQR.A
3	Beta-casein	288098	25.2	65	12 %	K.SPTIPFFDPQIPK.L
						K.VLPIPQQVVPYPQR.A
4	Lactotransferrin	16198359	78.3	333	8 %	R.YYGYTGAFR.C
						R.THYYAVAVVK.K
						R.SDTSLTWNSVK.G
						R.CLAENAGDVAFVK.D
						R.RSDTSLTWNSVK.G
						K.LRPVAAEVYGTER.Q
	Immunoglobulin kappa light chain VLJ region	21669409	29.8	118	17 %	R.TVAAPSVFIFPPSDEQLK.S
						K.DSTYSLSSTLTLSK.A
						K.VYACEVTHQGLSSPVTK.S
	Beta-casein	288098	25.2	106	28 %	K.SPTIPFFDPQIPK.L
						K.VLPIPQQVVPYPQR.A
						R.AVPVQALLLNQELLLNPTHQIYPVTQPLAPVHNPISV.-
	Immunoglobulin lambda light chain	33700	25.2	77	20 %	R.SYSCQVTHEGSTVEK.T
						K.YAASSYLSLTPEQWK.S
						K.AAPSVTLFPPSSEELQANK.A
5	Immunoglobulin kappa light chain VLJ region	21669357	29.5	381	34 %	K.LLIYWASTR.E
						K.DSTYSLSSTLTLSK.A
						K.SGTASVVCLLNNFYPR.E
						K.LYACEVTHQGLSSPVTK.S
						R.TVAAPSVFIFPPSDEQLK.S
						K.VDNALQSGNSQESVTEQDSKDSTYSLSSTLTLSK.A
	Alpha S1-casein	1359714	20.7	192	19 %	R.LQNPSESSEPIPLESR.E
						R.LNEYNQLQLQAAHAQEQIR.R
	Immunoglobulin lambda light chain	33700	25.2	139	14 %	K.YAASSYLSLTPEQWK.S
						K.AAPSVTLFPPSSEELQANK.A
	Beta-casein precursor	4503087	25.4	115	11 %	K.SPTIPFFDPQIPK.L
						K.VLPIPQQVVPYPQR.A
6	Beta-casein	288098	25.2	49	12 %	K.SPTIPFFDPQIPK.L
						K.VLPIPQQVVPYPQR.A
7	Alpha S1-casein	1359714	20.7	56	10 %	R.LNEYNQLQLQAAHAQEQIR.R
	Beta-casein	288098	25.2	50	12 %	K.SPTIPFFDPQIPK.L
						K.VLPIPQQVVPYPQR.A
8	Beta-casein	288098	25.2	38	12 %	K.SPTIPFFDPQIPK.L
						K.VLPIPQQVVPYPQR.A
9	Beta-casein	288098	25.2	81	12 %	K.SPTIPFFDPQIPK.L
						K.VLPIPQQVVPYPQR.A
	tenascin	37227	240.6	70	1 %	R.LEELENLVSSLR.E
	alpha S1-casein	1359714	20.7	65	10 %	R.LNEYNQLQLQAAHAQEQIR.R
10	Lactoferrin	186833	78.3	203	3 %	R.THYYAVAVVK.K
						K.LADFALLCLDGK.R
						K.NLLFNDNTECLAR.L
	Beta-casein	288098	25.2	38	6 %	K.VLPIPQQVVPYPQR.A

## Discussion

When mother’s own milk is not sufficient, donor milk is the first best choice for feeding term and preterm newborns, due to its well-recognized nutritional advantages with respect to formula milk [4–8]. DM should be obtained from established HMBs that follow specific guidelines for screening, storage, and handling procedures to optimize its composition while ensuring its safety for the recipient. [2, 10] All the milk arriving to the Human Milk Bank must be pasteurized. The ideal pasteurization process should consist of a phase of rapid heating followed by a phase of constant maintenance of the temperature and a final phase of rapid cooling. Pasteurization of the milk minimizes the risk of disease transmission via HM, inactivating most of the viral and bacterial contaminants. In addition, donors are screened in a similar way as for blood donation. No report has been published showing transfer of diseases through pasteurized DM, although milk may contain microorganisms. [2, 10]. Currently Holder Pasteurization (62.5 °C for 30 min) is the gold standard for milk processing in Human Milk Banks and is recommended by several guidelines as the optimal compromise between quality and microbiological safety [2, 10]. Several studies have already been performed to evaluate the effects of pasteurization on mother’s milk macronutrients; the related results are often discordant, especially concerning proteins [8, 20–22].

Our study observed a non-significant reduction of the total protein count following HoP of milk samples. This result is in line with the available literature: several studies reached similar conclusions using different analytical methods [20, 21]. Only Vieira et al. in 2011 found that there was a significant reduction in the mean protein concentrations, between the raw and post-pasteurization samples (reduction of 3.9 %). However, these samples were only analyzed with the use of a FT-IR infrared analyzer [22].

The original contribution of our study consists of the use of a semi-quantitative analytical method, GeLC-MS analysis. This technique allows to evaluate the protein profile of human milk, which is constituted by a complex array of biologically active proteins. Each sample was tested and compared under reducing and nonreducing conditions, to highlight the possible presence of protein-complexes due to disulfide bond formation in the pasteurization step. However this method is not able to evaluate the protein changes associated whit interaction between proteins and sugars, or proteins and lipid due to thermic treatments. No differences were observed between the electrophoretic profile of the same sample under reducing and nonreducing conditions, except for the region of high molecular weight, probably corresponding to the formation of high-mass complexes which do not run in the gel. Over 75 kDa, we observed more numerous and well separated bands under reducing conditions. The peculiarity in our data consists of the observation that no variation is present in 14 out of 20 samples (previous studies have detected a variation in all samples). It is also noteworthy that amongst the 6 samples presenting a modification in the protein levels, 5 derived from colostrum milk and only 1 from transitional milk. This finding is relevant from a clinical practice standpoint, since donor milk usually consists of mature milk (which, according to our data, did not show any variation).

Each band showing a variation was then identified with the corresponding protein(s). The bands of greatest interest are the ones of medium molecular weight (25–30 kDa) since they contain beta casein, alpha-casein, lactoferrin fragments and immunoglobulin light chains (Igκ and Igλ); the other band of interest (250 kDa) was identified as tenascin.

Tenascin is a homohexameric disulfide-linked glycoprotein and its expression decreases with lactation. This protein was investigated in recent studies for its antimicrobial properties in milk and Fouda et al. have hypothesized its ability to neutralize the HIV-1 virus via binding to the chemokine receptor site [23]. Tenascin has never been evaluated previously in human milk after pasteurization and we observed a reduction only in reduced samples of colostrum.

Beta-casein and alpha-casein are two important proteins of human milk. Only one previous study has investigated the effect of Holder pasteurization on these proteins using an electrophoretic analysis method and has shown a slight decrease in a single pooled sample [14].

IgA antibodies in breast milk provide passive immunity and sufficient protection against infections to neonates and preterm infants [24]. Lactoferrin is a bioactive protein that serves as an immunomodulatory factor [24]. Several studies revealed a number of functions of these molecules: i) IgA has specific antigen-targeted anti-infective action; ii) lactoferrin plays important roles in immunomodulation, iron chelation and antimicrobial action, and exhibits the anti-adhesive and trophic properties necessary for intestinal growth [24, 25]. Overall, regarding lactoferrin and IgA, our study is partially in agreement with the results reported in the literature, in which a decrease was found in all samples tested, in contrast to our findings. There might be two reasons for these differences: i) we did not use pooled milk (as done in several previous studies) but we have analyzed all milk samples individually; furthermore the same samples have been analyzed before and after pasteurization; ii) we have utilized a GeLC-MS analysis (semi-quantitative technique), instead of an immunohistochemical analytical techniques like the ELISA test. Additionally, there is a significant variability in previous studies concerning the retention of these proteins after HoP, which can be ascribed not only to heterogeneity in methods of human milk collection but also to treatment and storage before and after HoP [11–13].

## Conclusion

In conclusion, donor milk needs a heat treatment for a safety storage. Holder Pasteurization is the method recommended by all international guidelines for the constitutions of Human Milk Banks. Our data are in agreement with literature showing a small decrease in the total protein content of human milk after HoP. Nonetheless, samples obtained from different donors reacted differently to heat treatment, even if processed and stored using the same conditions: in particular, 30 % of the samples showed some differences in the protein profile after HoP, whereas 70 % of the samples showed no detectable differences. The detectable effects on the protein profile were mainly in colostrum samples; this variation is quite interesting from a clinical standpoint, since donor milk is mostly constituted by mature milk. The reasons for this apparent variability is not clear and would deserve further investigation. In this setting, future studies should be designed to investigate whether these differences are also confirmed by other techniques able to assess the protein changes due to thermic treatments including the interaction between proteins and sugars, or proteins and lipid with possible toxic derivatives.

## Abbreviations

1DE: one-dimensional electrophoresis
DM: donor human milk
GA: gestational age
HM: human milk
HMB: human milk banking
HMBA: Human Milk Banking Associations
HoP: holder pasteurization
IDA: information-dependent acquisition
MS: mass spectrometry
NEC: necrotizing enterocolitis
NICU: Neonatal Intensive Care Unit
NO-HoP: no-holder pasteurized
NQ: nutritional quality
SDS-PAGE: sodium dodecyl sulfate-poly acryl amide gel electrophoresis

## References

[1] American Academy of Paediatrics (2012). Breastfeeding and use of human milk. Pediatrics.

[2] Arslanoglu S, Bertino E, Tonetto P, Italian Association of Human Milk Banks (2010). Guidelines for the establishment and operation of a donor human milk bank. J Matern Fetal Neonatal Med.

[3] Agostoni C, Buonocore G, Carnielli VP, De Curtis M (2010). Enteral nutrient supply for preterm infants: commentary from the European Society of Paediatric Gastroenterology, Hepatology and Nutrition Committee on Nutrition. J Pediatr Gastroenterol Nutr.

[4] Boyd CA, Quigley MA, Brocklehurst P (2007). Donor breast milk versus infant formula for preterm infants: systematic review and meta-analysis. Arch Dis Child Fetal Neonatal Ed.

[5] Quigley MA, Henderson G, Anthony MY, McGuire W (2007). Formula milk versus donor breast milk for feeding preterm or low birth weight infants. Cochrane Database Syst Rev.

[6] McGuire W, Anthony MY (2003). Donor human milk versus formula for preventing necrotising enterocolitis in preterm infants: systematic review. Arch Dis Child Fetal Neonatal Ed.

[7] Quigley M, McGuire W (2014). Formula versus donor breast milk for feeding preterm or low birth weight infants. Cochrane Database Syst Rev.

[8] Arslanoglu S, Corpeleijn W, Moro G, ESPGHAN Committee on Nutrition (2013). Donor human milk for preterm infants: current evidence and research directions. J Pediatr Gastroenterol Nutr.

[9] Moltó-Puigmartí C, Permanyer M, Castellote AI, López-Sabater MC (2011). Effects of pasteurisation and high-pressure processing on vitamin C, tocopherols and fatty acids in mature milk. Food Chem.

[10] HMBANA. Milk Banking Guidelines. 2009 30-10-2010. Ref Type: Online Source. http://www.hmbana.org. Accessed 9 Feb.2011.

[11] Untalan PB, Keeney SE, Palkowetz KH, Rivera A (2009). Heat susceptibility of interleukin-10 and other cytokines in donor human milk. Breastfeed Med.

[12] Goelz R, Hihn E, Hamprecht K, Dietz K (2009). Effects of different CMV-heat-inactivation-methods on growth factors in human breast milk. Pediatr Res.

[13] Ewashuk JB, Unger S, O’Connor DL, Stone D (2011). Effect of pasteurization on selected immune components of donated human breast milk. J Perinat.

[14] Baro C, Giribaldi M, Arslanoglu S, Giuffrida MG, Dellavalle G, Conti A (2011). Effect of two pasteurization methods on the protein content of human milk. Front Biosci.

[15] Playford RJ, Macdonald CE, Johnson WS (2000). Colostrum and milk-derived peptide growth factors for the treatment of gastrointestinal disorders. Am J Clin Nutr.

[16] Bradford MM (1976). A rapid and sensitive method for the quantitation of microgram quantities of protein utilizing the principle of protein-dye binding. Anal Biochem.

[17] Neuhoff V, Arold N, Taube D, Ehrhardt W (1988). Improved staining of proteins in polyacrylamide gels including isoelectric focusing gels with clear background at nanogram sensitivity using Coomassie Brilliant Blue G-250 and R-250. Electrophoresis.

[18] Spertino S, Cipriani V, De Angelis C, Giuffrida MG (2012). Proteome profile and biological activity of caprine, bovine and human milk fat globules. Mol Biosyst.

[19] Bona E, Marsano F, Cavaletto M, Berta G (2007). Proteomic characterization of copper stress response in Cannabis sativa roots. Proteomics.

[20] Silvestre D, Miranda M, Muriach M, Almansa I (2008). Antioxidant capacity of human milk: effect of thermal conditions for the pasteurization. Acta Paediatr.

[21] Hamprecht K, Maschmann J, Müller D, Dietz K (2004). Cytomegalovirus (CMV) inactivation in breast milk: reassessment of pasteurization and freeze-thawing. Pediatr Res.

[22] Vieira AA, Soares FVM, Pimenta HP, Abranches AD (2011). Analysis of the influence of pasteurization, freezing/thawing, and offer processes on human milk’s macronutrient concentrations. Early Hum Dev.

[23] Fouda GG, Jaeger FH, Amos JD, Ho C (2013). Tenascin-C is a innate broad-spectrum, HIV-1-neutralizing protein in breast milk. Proc Natl Acad Sci U S A.

[24] Lawrence RA, Lawrence RM (2005). Breastfeeding.

[25] Hamosh M (2001). Bioactive factors in human milk. Pediatr Clin North Am.

